# Antibacterial Activity of Rose Bengal Entrapped in Organically Modified Silica Matrices

**DOI:** 10.3390/ijms23073716

**Published:** 2022-03-28

**Authors:** Yanna Gurianov, Michael Meistelman, Yael Albo, Marina Nisnevitch, Faina Nakonechny

**Affiliations:** Department of Chemical Engineering, Ariel University, Kyriat-ha-Mada, Ariel 4070000, Israel; yannag@ariel.ac.il (Y.G.); michaelme@ariel.ac.il (M.M.); marinan@ariel.ac.il (M.N.)

**Keywords:** Rose Bengal, photosensitizers, sol-gel, modified silica, antibacterial surfaces

## Abstract

Photosensitizers (PSs) are known as powerful antibacterial agents that are activated by direct exposure to visible light. PSs can be noncovalently entrapped into the silica gel network for their controlled release into a contaminated area. The immobilization of PS-containing gel matrices on a polymer support expands their possible applications, such as antibacterial surfaces and coatings, which can be used for the disinfection of liquids. In the current study, we report the use of Rose Bengal (RB) incorporated into organically modified silica matrices (RB@ORMOSIL matrices) by the sol-gel technique. The RB matrices exhibit high activity against Gram-positive and Gram-negative bacteria under illumination by white light. The amount and timing of solidifier addition to the matrix affected the interaction of the latter with the RB, which in turn could affect the antibacterial activity of RB. The most active specimen against both Gram-positive and Gram-negative bacterial cells was the RB6@ORMOSIL matrix immobilized on a linear low-density polyethylene surface, which was prepared by an easy, cost-effective, and simple thermal adhesion method. This specimen, RB6@OR@LLDPE, showed the low release of RB in an aqueous environment, and exhibited high long-term antibacterial activity in at least 14 rounds of recycled use against *S. aureus* and in 11 rounds against *E. coli*.

## 1. Introduction

According to the WHO, hundreds of millions of people suffer from healthcare-associated infections (HAIs) each year. One of the most common sources of HAIs is contaminated polymeric medical surfaces and devices [1]. Since polymers do not possess antimicrobial activity, there is a need to develop antibacterial materials for medical applications to thus prevent the transmission of HAIs [2].

There are several approaches to preparing polymers with antibacterial activity by dip-coating. They include immersing cotton fabrics in colloidal suspensions [3]; covalent grafting, in which antibacterial agents are covalently attached to the surface of polymers, through a two-step argon plasma treatment or through chemical reactions [4,5]; a layer-by-layer technology (LBL), which creates LBL antibacterial films by adsorption of an electrolyte on the substrate surface [6,7]; preparation of antibacterial films using material blending methods in mixing different polymers [8], such as cationic amphiphilic block co-polymers with polystyrene [9]; nanoengineered polymers, utilizing, for example, inorganic or organic nanostructured solid templates [10]; and using organically modified silica (ORMOSIL) coatings prepared by the sol-gel technique [11]. The latter process is a simple and effective method that gives antibacterial properties to existing polymeric surfaces by coating them with ORMOSIL-incorporated antibacterial agents. In recent years, this technology has been shown to be a very promising tool for the entrapment of active molecules [12]. The application of an antimicrobial sol-gel coating can inhibit and eradicate bacteria, fungi or other microorganisms. For instance, Goh et al. focused on silicate-based bioactive glass (BG) nanoparticles and showed that Cu-doped BG exhibited a prolonged release of ions, suggesting it as a good candidate for long-term use in antibacterial material [13]. These biomaterials can be applied in the bone regeneration field as fillers, coatings for implants, and scaffolds in tissue engineering [13]. Trabelsi et al. prepared SilverSil (ORMOSIL doped with Ag^0^ nanoparticles), which demonstrated an outstanding antimicrobial activity against *S. aureus* and *E. coli* cells. This material can serve as an antibacterial coating in lifesaving applications in hospitals, schools, and industrial and commercial environments [14].

Antibacterial agents can be noncovalently entrapped into the gel network for their controlled release into the contaminated area. The antimicrobial active species can be added at different stages of the sol-gel process. Incorporation is made possible by mixing them with the sol-gel precursor solution in the hydrolysis stage; or alternately, the addition of the agents can be performed when a sol is obtained [15].

Photosensitizers (PSs) are known as powerful antibacterial agents that are activated by direct exposure to visible and near infrared light [16,17,18]. This procedure is called photodynamic antimicrobial chemotherapy (PACT), which is a powerful tool for killing Gram-positive and Gram-negative bacteria [19,20]. PSs are molecules with a system of conjugated double bonds [21]. Under illumination, these molecules are excited, and can either exchange an electron with other substances (Type I mechanism) or transfer their excitation energy to dissolved molecular oxygen (Type II mechanism). In the Type I mechanism, PS molecules react with bio-organic molecules, producing active free radicals and radical ions of the PS or another organic substrate, which further react with oxygen, producing reactive oxygen species, ROS (peroxides, superoxide ions and hydroxyl radicals). The produced ROS irreversibly alter the vital cell constituents, resulting in lethal damage [22]. In the Type II reaction, the PSs typically interact with triplet oxygen species upon irradiation with visible light to produce ROS, such as singlet oxygen ^1^O_2_, hydroxyl radical OH•, superoxide anion O^2−^ and hydrogen peroxide H_2_O_2_ [23]. Rose Bengal (RB) is an anionic water-soluble xanthene dye with a characteristic pink-red color, and is known as an efficient PS showing antibacterial [24], antifungal [25] and anticancer [26] activity [17,21]. RB is reported to participate in the Type II reaction [27]. Due to the high efficiency of RB as a PS, and the high versatility of the sol-gel process that enables one to obtain a material with tailored properties, we decided to prepare RB-incorporated ORMOSIL matrices.

Previous works that examined the RB-silica systems focused on covalently binding the RB to the surface of silica nanoparticles (NPs). For instance, Martins et al. (2015) [28] covalently conjugated RB to amino functionalized mesoporous silica nanoparticles, and the effects of RB loadings on singlet oxygen generation were studied. Guo et al. (2010) [29] investigated the inactivation of bacteria by RB-decorated silica NPs. A three-step preparation procedure was used. Silica nanoparticles were prepared, their surface was functionalized with amine groups, and then RB-dye molecules were covalently conjugated to the silica surface.

The present work aimed to incorporate RB into ORMOSIL matrices using a one-pot synthesis procedure by applying the sol-gel technique, and then examine the activity of the samples against Gram-negative and Gram-positive bacteria, under illumination with white light and in the dark. The most active specimen was chosen for immobilization onto polyethylene in order to produce an antibacterial surface.

## 2. Results and Discussion

### 2.1. Characterization of RB@ORMOSIL Matrix and RB6 Immobilized onto LLDPE

To evaluate the effects of synthesis parameters on the surface area and the pore volume of the matrices, N_2_ adsorption–desorption experiments were conducted. Samples BL1, RB4 and RB6 were chosen for the analysis, as these specimens were prepared by various approaches. The results are presented in Table 1. As can be seen, all the samples demonstrated a mesoporous nature, with pore diameters between 2 and 50 nm [30,31]. BL1, which did not contain RB, served as a blank reference sample. RB4 contained an amount of APTES 2.5 times greater than RB6. The decreases in the surface areas of RB4 and RB6, compared to BL1, were probably a result of RB incorporation into the host matrix. However, the average pore diameters and the average pore volumes were very close for all three samples (Table 1).

**Figure 1:**
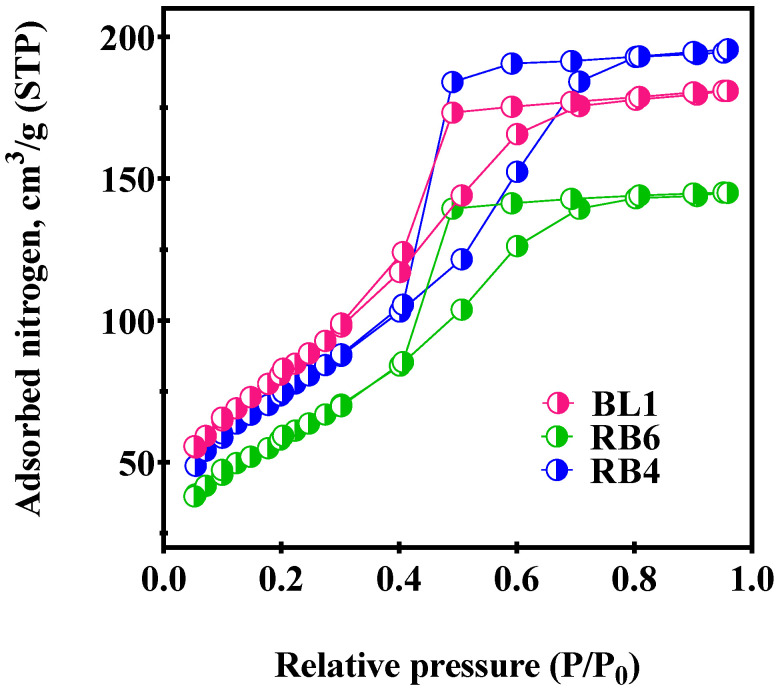
Nitrogen adsorption–desorption isotherms of BL1, RB4 and RB6.

The results of N_2_ adsorption and desorption by the samples BL1, RB6 and RB4 are shown in Figure 1. In all cases, the process occurred according to the Type IVA adsorption–desorption isotherm pattern [31]. Hysteresis loops in the sample isotherms exhibited H2A loops typical for silica gels [31], indicative of interconnected networks of ink bottle-shaped pores [32,33,34] that may contribute to diffusion-controlled reactions.

**Figure 2:**
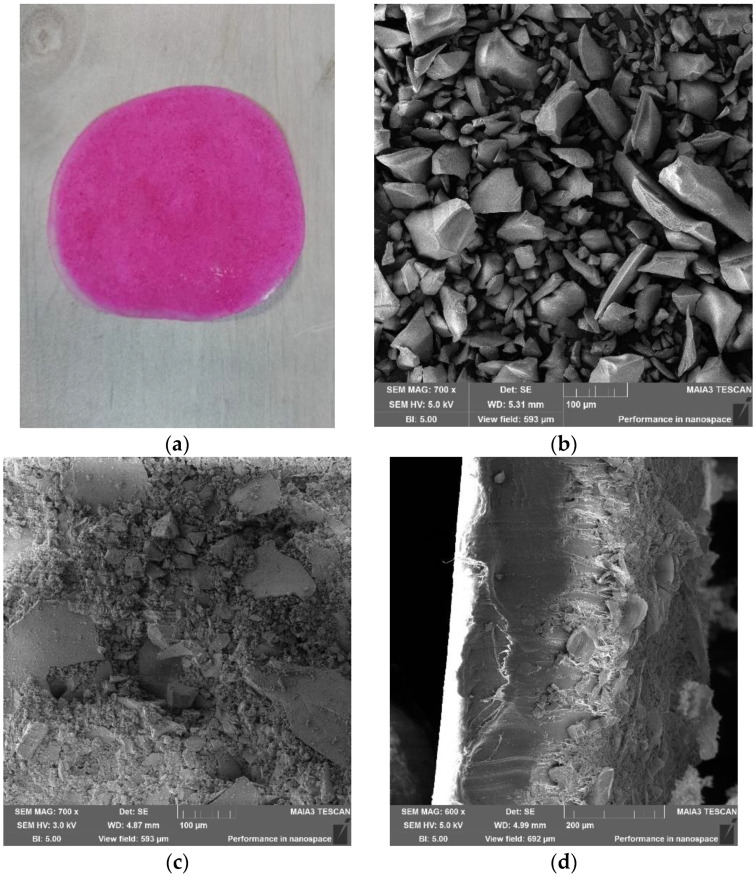
Photographic image of the specimen RB6@OR@LLDPE (RB6 immobilized onto LLDPE by thermal adhesion) (**a**); SEM micrographs of surfaces of RB6 powder (**b**), and of RB6@OR@LLDPE (**c**); a cross-section of RB6@OR@LLDPE (**d**).

The samples of dried and crushed RB6 matrix in free form, and immobilized onto LLDPE (specimen RB6@OR@LLDPE), were examined by scanning electron microscopy (SEM) (Figure 2). In addition, the RB6@OR@LLDPE specimen was photographed, and it can be seen that the polymer surface of the RB6@OR@LLDPE was evenly covered with RB entrapped in the ORMOSIL matrix, exhibiting the characteristic pink–red color (Figure 2a). It should be mentioned that the RB6 matrix included 5.94 µmol of RB per g of the matrix (Table 2), and the RB6@OR@LLDPE specimen was loaded by 89.4 nmol RB per cm^2^ of the polymer surface. The SEM micrographs show that the RB6 matrix (Figure 2b) looked like a typical ORMOSIL matrix powder, while the polymeric surface of RB6@OR@LLDPE was coated with pressed RB6 matrix powder (Figure 2c). The cross-section image of RB6@OR@LLDPE (Figure 2d) presents the RB6 matrix coating layer on the right side of the image, while the polymer itself can be seen on the left side. The thickness of the obtained specimen was 265 ± 10 µm.

### 2.2. FTIR Examination of Matrices

**Figure 3:**
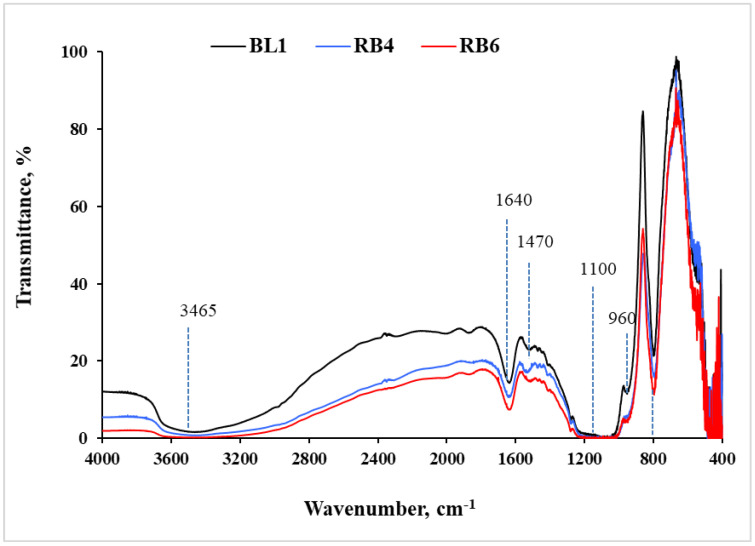
FTIR spectra of BL1, RB4 and RB6 matrices.

The identification of functional groups in the BL1, RB4 and RB6 matrices was performed by FTIR spectroscopy (Figure 3). For all samples, absorption bands were detected at around 3465 cm^−1^, 1640 cm^−1^, 1470 cm^−1^, 1100 cm^−1^, 960 cm^−1^ and 800 cm^−1^. The absorption bands at 3465–3468 cm^−1^ and at 950–968 cm^−1^ were assigned to the stretching vibrations of the OH groups in the silica matrix. The band at 1635–1642 cm^−1^ was related to water molecule deformation. The absorption signals at 1086–1181 cm^−1^ and 787–798 cm^−1^ corresponded to the asymmetric and symmetric stretch of Si–O–Si bonds, respectively. The absorption band at 1470–1532 cm^−1^ was attributed to the stretching vibration of the C–H bond [35,36]. Since there were no significant differences in the spectra of blank matrices and specimens containing RB, it may be concluded that the entrapment of RB into the ORMOSIL matrices did not affect the internal structure of the matrix.

### 2.3. Spectroscopic Analysis of RB@ORMOSIL

**Figure 4:**
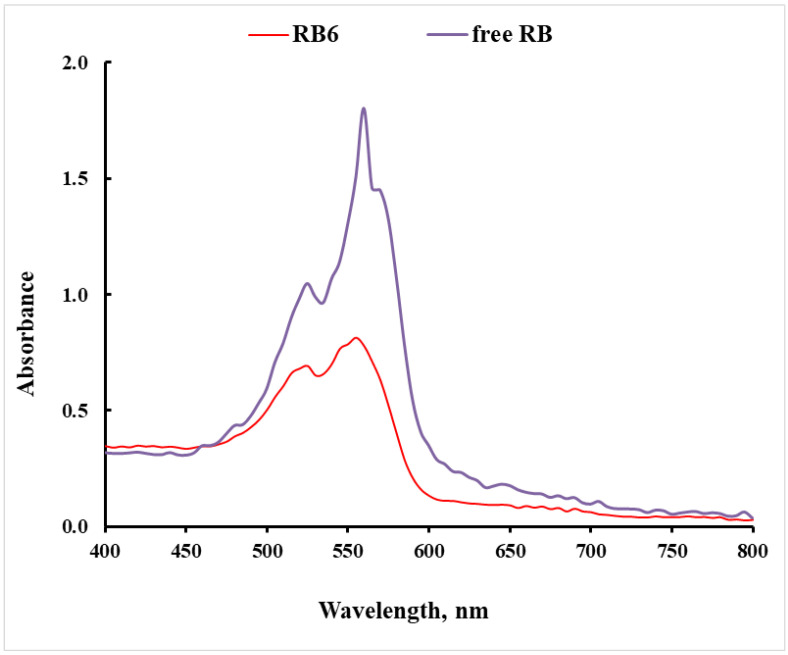
Visible spectra of free RB and RB6 matrix.

To test if RB keeps its spectral properties when incorporated into the RB@ORMOSIL matrices, the visible spectrum of RB6 was compared to that of free RB. As can be seen in Figure 4, after the incorporation of RB in a matrix, the λ_max_ of RB underwent a blue shift from 560 to 550 nm. This shift can be explained by electrostatic interaction with amino groups of the matrix. A similar phenomenon was observed when RB was examined in the presence of arginine [37].

### 2.4. Leaching of RB from RB@ORMOSIL and RB@OR@LLDPE Specimens

After the preparation of the RB@ORMOSIL matrices, the wet gel was dried at room temperature, then crushed into a powder and washed several times with measured amounts of saline solution, in order to remove the non-entrapped RB. Table 2 presents the results of the experiment and the total RB leakage into a saline solution for all washes (%). The RB leakage was highest in the case of RB2, and lowest in RB3. For other matrices, the leakage did not exceed 46%. The actual loading of RB in matrices after the washing is shown in Table 2. The loading of RB in matrices RB1–RB6 ranged between 4.67 and 7.83 µmol/g. The observed data scattering was due to different amounts of leached RB and slight dispersion in the masses of the obtained matrices.

**Figure 5:**
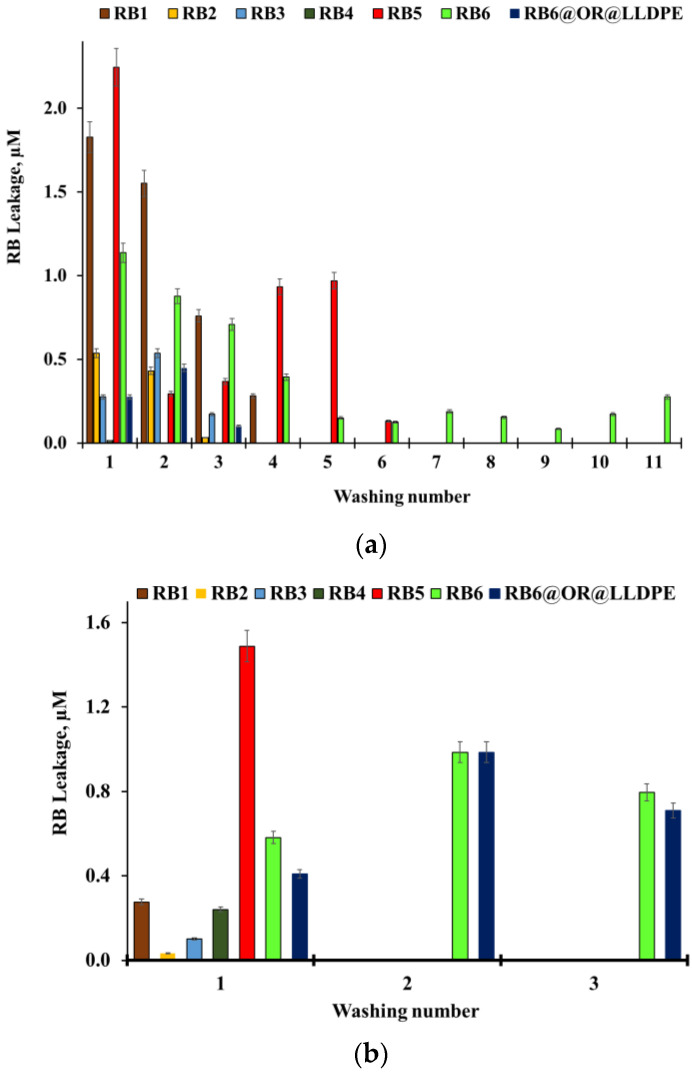
Leaching of RB from RB1, RB2, RB3, RB4, RB5, RB6 and RB6@OR@LLDPE into bacterial suspensions of *E. coli* in saline under illumination with a white luminescent lamp (fluence rate 39.5 mW cm^−2^) (**a**) and in the dark (**b**) after a number of washings.

In addition, the leaching of RB from the RB@ORMOSIL matrices into the saline solution in the presence of bacterial cells was studied under illumination and in the dark. The matrix specimens were placed in Petri dishes that contained fresh bacterial suspensions, which were changed several times. Each washing was examined for RB leaching. The presence of *S. aureus* cells in the saline did not affect the leaching of RB, and in all cases the RB absorption did not exceed 0.01 at 550 nm (data not shown). Addition of the *E. coli* suspension into the saline caused a slight release of RB from RB@ORMOSIL matrices (Figure 5). Interestingly, RB leakage was a bit higher under illumination (Figure 5a) than in the dark (Figure 5b); however, in all cases, the concentration of leaked RB did not exceed 2.2 µM.

### 2.5. Antimicrobial Activity of RB@ORMOSIL and RB@OR@LLDPE Specimens

**Figure 6:**
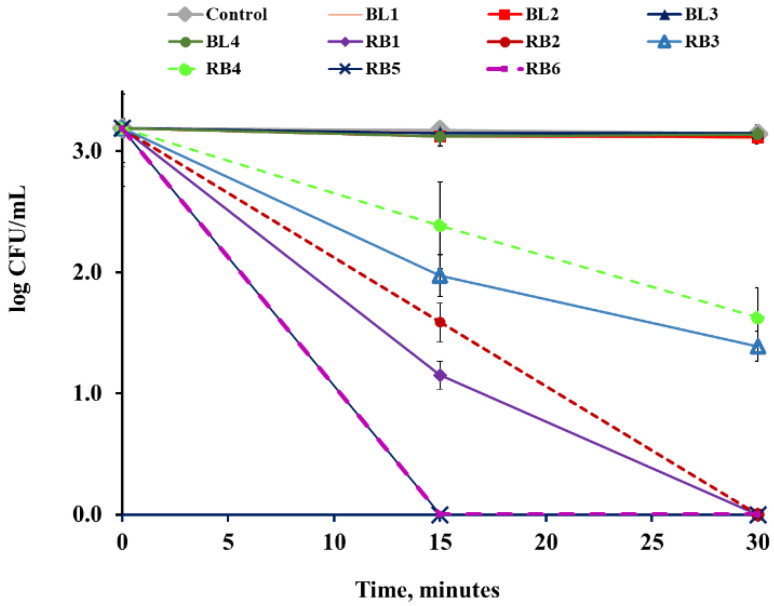
Activity of BL1–BL4 and RB1–RB6 against *E. coli* under illumination with a white luminescent lamp at a fluence rate of 39.5 mW cm^−2^ at 25 °C. Control—untreated bacterial cells.

The blank and RB-loaded RB@ORMOSIL matrices were dispersed on the surfaces of sterile Petri dishes, as described in Section 3.5, and tested for antimicrobial activity against *S. aureus* and *E. coli* under white light illumination. All the RB-loaded matrices eradicated *S. aureus* after 5 min, whereas all the blank matrices showed no antibacterial activity (data not shown). In the case of *E. coli,* only two matrices, RB5 and RB6, eradicated all the cells after 15 min (Figure 6). The RB1 and RB2 matrices after 15 min decreased the cell concentration by 2 log_10_, and the total eradication of bacteria was achieved after only 30 min. The RB3 and RB4 matrices showed only moderate activity; after 30 min the cell concentration dropped by only 1.5 log_10_. The difference in the antibacterial activity of the matrices can be explained by different compositions of the matrices; RB3 and RB4 contained less RB relative to APTES, compared to the rest of the specimens (please see Section 3, Table 3). As expected, blank matrices did not exhibit any antibacterial activity against either of the bacteria cells (Figure 6). To exclude the effect of possible cell overheating under illumination, the temperature was monitored during the experiments. The temperature in the cell suspensions never exceeded 27 °C.

**Figure 7:**
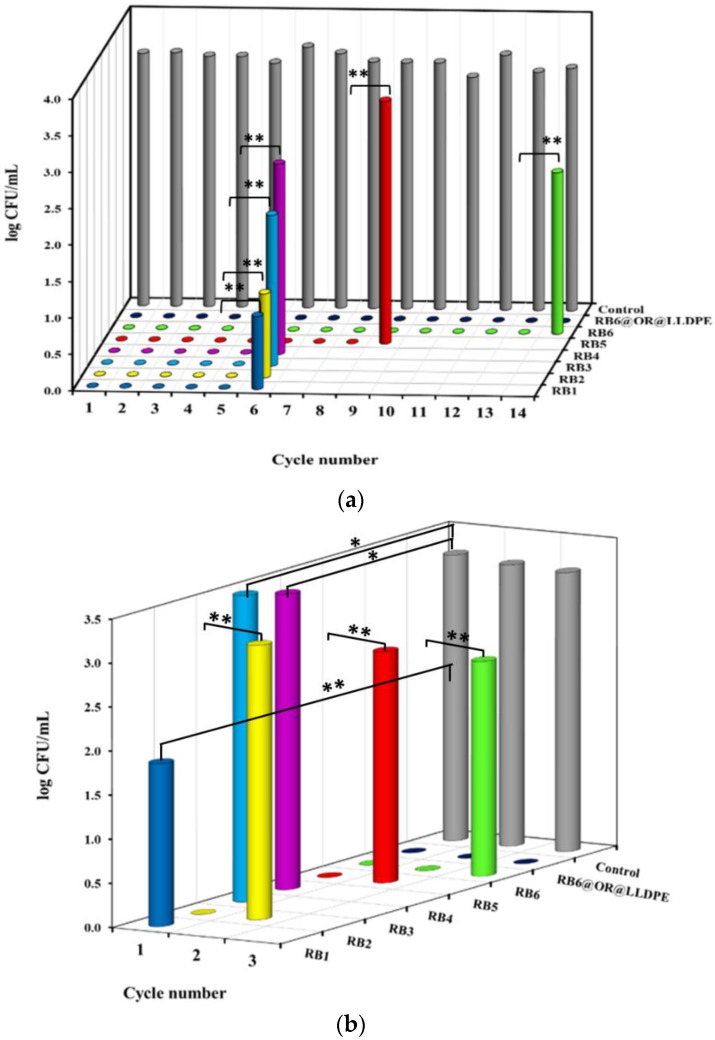
Antibacterial activity of RB1–RB6 and RB6@OR@LLDPE against *S. aureus* under illumination at a fluence rate of 39.5 mW cm^−2^ (**a**), and in the dark (**b**), at 25 °C for 5 min. Control—untreated bacterial cells. Figures at x-axis show the number of usage cycles. Control—*S. aureus* after 5 min incubation. Results with no statistical difference are marked by one asterisk and statistically different results (*p* < 0.05) are marked by two asterisks.

The RB@ORMOSIL and RB6@OR@LLDPE samples were also tested for their ability to eradicate bacteria when reused. This series of experiments was performed using a cell strainer-filter on which the specimens were fixed, in order to enable their transfer from one Petri dish to another containing fresh suspension of bacterial cells, for a number of repeated uses (cycles of usage). The results of the experiments are presented in Figure 7. In experiments with *S. aureus,* RB1–RB4 samples continued to kill bacteria for 5 cycles of usage, RB5 for 8 cycles, and RB6 for 13 cycles. The RB6@OR@LLDPE was active for at least 14 cycles (Figure 7a). In the dark, the specimens were either completely inactive (RB1, RB3 and RB4), or active only in the first one or two cycles, and then lost their antibacterial activity (RB2, RB5 and RB6) (Figure 7b).

**Figure 8:**
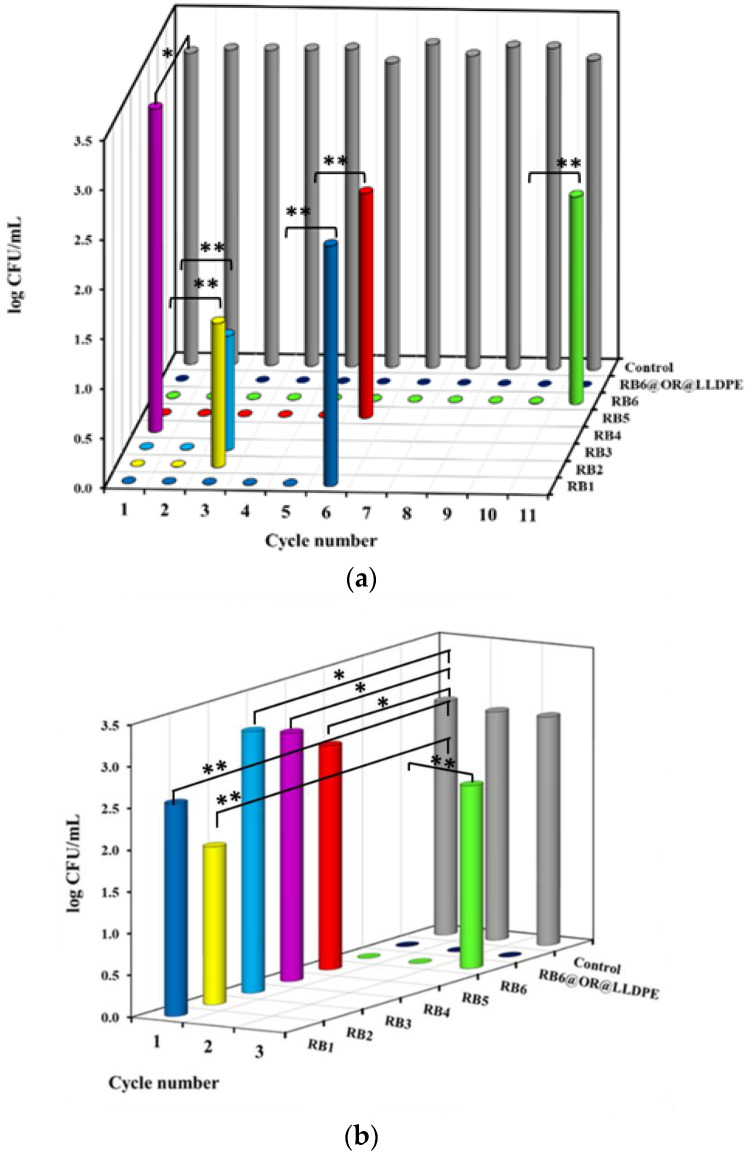
Antibacterial activity of RB1, RB2, RB3, RB4, RB5, RB6 and RB6@OR@LLDPE against *E. coli* under light illumination at fluence rate of 39.5 mW cm^−2^ (**a**), and in the dark (**b**), at 25 °C for 75 min. Control—untreated bacterial cells. Figures on the x-axis show the number of cycles. Control—*E. coli* after 75 min incubation. Results with no statistical difference are marked by one asterisk and statistically different results (*p* < 0.05) are marked by two asterisks.

In the experiments with *E. coli,* the immobilized matrices RB1 and RB5 were active against bacterial cells for five cycles, while RB2 and RB3 eradicated *E. coli* cells for two cycles only. RB4 was inactive against the *E. coli* cells. The most active matrix was RB6, which eradicated bacterial cells for 10 cycles (Figure 8). The RB6@OR@LLDPE sample totally eradicated the *E. coli* cells for at least 11 cycles (Figure 8).

All the matrices demonstrated higher antibacterial activity against Gram-positive *S. aureus* than against Gram-negative *E. coli*, which can be seen from the shorter eradication time and higher recycling numbers in the former bacteria. This phenomenon was also observed previously by us [38,39,40] and by others [41].

In the experiments in the dark, the antibacterial activity of RB@ORMOSIL matrices against *E. coli* was also less than that against *S. aureus* (Figure 7b and Figure 8b). Samples RB1–RB5 were completely inactive against *E. coli*, whereas RB6 was active for two cycles. The RB6@OR@LLDPE specimen was active in the dark against both bacteria in at least three cycles. The above data show that the most active specimen against both bacteria cells was the RB6 immobilized on the LLDPE surface.

The amount of APTES added to the matrix affects the number of amine groups on the sol-gel wall, and therefore, the interaction of the matrix with the RB. The latter interaction can in turn affect the antibacterial activity of the RB; this may explain the differences in the activity of the different matrices. Due to the porosity of the matrices, most of the entrapped RB may not be available for contact with bacterial cells. The stage during the sol-gel process at which RB and APTES are added may therefore influence the availability of RB for antibacterial activity. We showed in this work that for the highest activity and minimal leakage from the matrices, the recommended preparation follows that of the RB6 sample, where RB was added parallel to the addition of the APTES solidifier. The most active specimen against both Gram-positive and Gram-negative bacterial cells was RB6 immobilized on the LLDPE surface. This specimen’s high activity was due to the timing of the addition of RB with the gel synthesis, resulting in the even distribution of the RB6 matrix on the polymeric surface.

The immobilization of photosensitizers on polymers expands their possible applications by allowing continuous or repeated use. Such polymers can serve as antibacterial surfaces and coatings, and can also be used for the disinfection of liquids. These possibilities have aroused interest in studying the properties of PSs immobilized onto polymers against various bacteria.

For example, the photosensitizer RB immobilized in polystyrene, polycarbonate, and polymethyl methacrylate was shown to be effective in killing *S. aureus* under moderate illumination. At the same time, the antibacterial activity of polymers with immobilized RB was related to the polymer structure. It was suggested that the porous surface of polystyrene promoted the better adhesion of bacterial cells to the polymer than the smooth surfaces of polycarbonate and polymethyl methacrylate, resulting in a higher antibacterial activity of RB-polystyrene, compared to the two other polymers [42]. Photo-antimicrobial conjugates of RB with cationic polystyrene showed effective photodynamic inactivation of *Staphylococcus aureus*, *Escherichia coli*, *Enterococcus faecalis* and *Pseudomonas aeruginosa* bacteria, as well as a moderate reduction in the population of the yeast *Candida albicans* [43,44]. In addition, RB immobilized in polystyrene showed high antibacterial activity when illuminated with visible white light, destroying Gram-positive *S. aureus*, Gram-negative *E. coli* and coliform bacteria in wastewater in a continuous mode. The concentration of bacteria in flow reactors with immobilized RB decreased by two to five orders of magnitude [39]. The porous structure of the matrices used in the present work is another factor that possibly enhances their effectiveness.

The RB immobilized in polyethylene and polypropylene showed a good ability to reduce the concentrations of *S. aureus* and *E. coli,* in periodic and continuous modes under illumination with a white luminescent lamp, up to their total eradication [40]. In this study, the effect of immobilized PS loading and bacterial concentration on the rate of cell eradication was studied. The immobilization of RB and other PSs in talc was also effective, with the mechanochemical treatment proposed in this study slowing PS leakage from the talc support by a factor of 10–30, compared to untreated mixtures. Immobilized photosensitizers were active against Gram-positive and Gram-negative bacteria [40].

Recently, the immobilization of RB in silicone was proposed, and the possibility of reusing the polymer to kill *S. aureus* under ultrasonic activation was demonstrated. In its two first uses, the polymer exhibited the same activity, reducing cell concentration by 2.5 log_10_ in 1 min. However, in the third application, the polymers were almost inactive, apparently due to RB leaching from the silicone substrate under ultrasound [24]. In the present work, the immobilized RB remained active and continued to kill bacteria under light activation for 2–13 cycles in the case of RB@ORMOSIL matrices, and even longer, for 11–14 cycles, in the case of RB6@OR@LLDPE (Figure 7a and Figure 8a).

It is important to mention the dark toxicity of RB. Antibacterial effects of RB against various microorganisms in the absence of illumination were noted by us [24] and others [45,46].

In all reported cases, this activity was much lower than under the effect of light. The phenomenon was also observed in this study. The RB6@ORMOSIL matrix destroyed *S. aureus* and *E. coli* cells in the dark for two cycles, and RB6@OR@LLDPE for at least three cycles (Figure 7b and Figure 8b). The rest of the matrices were less active, or not active at all, in the dark (Figure 7b and Figure 8b). At the same time, it should be noted that the dark toxicity of RB does not reduce the possibility of using the coatings suggested here; on the contrary, they are expected to exhibit a certain rate of antibacterial activity even in the absence of illumination.

## 3. Materials and Methods

### 3.1. Materials

Tetraethylorthosilane (TEOS, >99%), methyltrimethoxysilane (MTMOS, 97%), (3-aminopropyl)triethoxsylane (APTES, 99%), and linear low-density polyethylene (LLDPE) were purchased from Sigma-Aldrich^®^ (St. Louis, MO, USA). LCMS-grade ethanol (EtOH) was purchased from Bio-Lab Ltd. (Jerusalem, Israel). Sodium chloride (analysis grade) and HCl (37%) were purchased from Merck Millipore Ltd. (Carrigtohill, Ireland). RB (90% purity) and nujol oil were purchased from Alfa Aesar (Heysham, UK).

### 3.2. RB@ORMOSIL Synthesis via the Sol-Gel Route

RB@ORMOSIL matrices were prepared using the two-step acid/base sol-gel synthesis route. The first synthesis stage was common to all ten prepared matrices: 4.1 mL of water containing 100 µL of HCl was added dropwise into a premixed solution containing 813 µL of MTMOS, 11.45 mL of TEOS and 13.26 mL of EtOH. The resulting mixture was stirred for 15 min at 200 rpm. APTES was added in portions of 600 µL (RB1, RB5 and RB6), 900 µL (RB2), 1200 µL (RB3) or 1500 µL (RB4); 5 mL of RB in aqueous solution (8.6 mg/mL) was added at different stages of the synthesis: immediately after the addition of APTES (RB1, RB2, RB3, RB4), before its addition (RB5), or parallel to its addition (RB6) (Table 3). The mixtures were stirred vigorously. Blank matrices BL1, BL2, BL3 and BL4 were prepared following the same procedures, except that 5 mL water was added instead of the RB solution (Table 3). The wet gel was kept for one month in the dark for aging and drying at room temperature. The obtained solid matrices were then crushed with a mortar and pestle into a powder, and washed several times with 1 L saline solution until transparent washings were obtained. The washed matrices were dried for another 14 days.

### 3.3. Thermal Adhesion of RB@ORMOSIL onto the LLDPE Polymer

The thermal adhesion of RB6@ORMOSIL onto the LLDPE polymer was performed as described by us earlier for the immobilization of copper nanoparticles onto LLDPE [47]. In brief, 1 g LLDPE pellets were melted at 130 °C using the maximal pressure of the heat-press machine under 450 kg_f_ for 3 min. Then, 0.5 g of the crushed matrix was dispersed on the molten polymer and slightly pressed for 20 s. The specimens were cooled to room temperature. The thickness of the specimens was measured with a digital 150 mm caliper (Roher^®^, Ramla, Israel). The obtained specimen was designated as RB6@OR@LLDPE.

### 3.4. Bacterial Growth

Cultures of Gram-positive *S. aureus* (ATCC 11522) and Gram-negative *E. coli* (ATCC 9723e) (ATCC, Manassas, Virginia) were grown in brain–heart infusion agar (BH) and Luria Bertani agar (LB) (Himedia^®^, Mumbai, India), respectively, for 24 h. The inoculum was transferred to a corresponding broth medium, grown at 37 ± 1 °C, and incubated under shaking at 150 rpm until reaching OD_660nm_ (optical density) = 0.3. The bacterial suspensions were diluted with sterile saline to a final concentration of 10^3^ cells·mL^−1^.

### 3.5. Antimicrobial Activity Test

First, the antibacterial activity of 0.5 g free-form matrices was tested. Second, new specimens of 0.5 g matrices were placed on a 40 µm pore-sized cell strainer (Alex Red Ltd., Ha-Tuv, Israel) to evaluate the reusability of the specimens against bacteria. Finally, a 1 g sample of LLDPE-coated matrix was tested. All specimens were tested as follows: 25 mL of bacterial suspension at a concentration of 10^3^ cells·mL^−1^ in sterile saline was placed in a 90 mm sterile Petri dish with a specimen (one specimen per dish). Then, the specimens were incubated at 25 ± 1 °C under shaking at 100 rpm in the dark or under a white luminescence lamp emitting light between 360 and 700 nm [38] (ORSAM, model L18W/765, cool daylight, with a fluence rate of 39.5 mW cm^−2^, Munich, Germany), for periods of 5 min for *S. aureus* and 75 min for *E. coli*. The light intensity was measured with an LX-102 light meter (Lutron, Taipei, Taiwan). The distance between the lamp and Petri dishes was 24 cm. Temperature in the cell suspensions was monitored during the experiments.; 100 µL samples of suspension from the specimens were distributed onto BH or LB agar plates, for *S. aureus* and *E. coli*, respectively. The plates were incubated overnight at 37 ± 1 °C in the dark, and the bacterial colony-forming units (CFU) were counted using a Scan 500 colony counter (Interscience, Saint-Nom-la-Bretèche, France).

### 3.6. Testing RB Leakage from RB@ORMOSIL Matrices into Saline Solution and Bacterial Suspensions

The leaching of RB from matrices was tested after the latter were dried and crushed, and tested again after incubation with bacterial suspensions. The former examination was carried out as follows: 1000 mL of saline was added several times to the powdered matrices, and suspensions were stirred at 120 rpm with a magnetic stirrer for 30 min. The solution was separated from the powder using a Buchner funnel supplied with a micro-glass fiber paper, 70 mm in diameter (Munktell Ashltrom Corporation, Helsinki, Finland). The concentration of RB released into the washing solution was determined by measuring the absorption at 550 nm, using a Genesys 10S UV-VIS spectrophotometer (Thermo Fisher Scientific Inc., Waltham, MA, USA). Testing for RB released from the matrices onto the cell strainer or polymer was performed in the samples taken for the antibacterial tests. The bacterial suspension was sampled by 1.0 mL aliquots at 5 and 75 min for *S. aureus* and *E. coli*, respectively, and filtered through Millex^®^-GV membranes with a 0.22 µm pore size (Merck Millipore Ltd., Carrigtohill, Ireland). The absorption of the sample was then measured as mentioned above.

### 3.7. FTIR Analysis

The infrared spectra were measured using the KBr pellet technique, by thoroughly mixing 10 mg of powdered matrix sample with 0.2 g of KBr, and pressing at 5 ton_f_ using a hydraulic press (Carver^®^ Inc., Wabash, IN, USA). The samples were analyzed by an FTIR-4600 spectrometer (Jasco Corporation, Tokyo, Japan) at room temperature in the 5000–400 cm^−1^ range at an operation number of 32 scans, a resolution of 2.0 cm^−1^, and a scanning interval of 1 cm^−1^.

### 3.8. Spectroscopic Analysis

Visible spectra of free RB and RB6 matrix were measured by a method proposed by Trabelsi et al., 2020 [14], and Meistelman et al., 2021 [48]. Then, 0.2 g specimens were mixed with 4 drops of nujol oil using mortar and pestle. The obtained pastes were spread evenly onto a 0.9 cm × 8 cm piece of weighing paper and fixed vertically in the 1.0 cm quartz cuvette. Absorbance spectra of the samples were registered using Varian Cary UV Bio 50 (Varian Australia Pty Ltd., Mulgrave, Australia) in a dual-beam mode.

### 3.9. BET Analysis

The BET measurements for the specific surface area, pore volume, and pore size distribution were taken using a Nova 3200e Quantachrome analyzer (Boynton Beach, FL, USA). Before the analysis, the samples were subjected to degassing at 120 °C under a vacuum for 2 h. The surface area was calculated from the linear part of the BET plot. The pore size distribution was estimated using the Barrett–Joyner–Halenda (BJH) model and the Halsey equation [30], whereas the pore volume was measured at the P/P_0_ = 0.9947 single point.

### 3.10. SEM Analysis

Imaging of RB6 powder surfaces and cross-sections of immobilized RB6 was performed with an SEM microscope (Tescan MAIA3, Triglav™, Brno, Czech Republic). The samples were placed on a carbon tape and covered with a 10 nm carbon layer using a Q150T ES Quorum coater (Quorum Technologies Ltd., Laughton, UK) under a sputter current of 12 mA for 30 s. SEM measurements were performed at operating voltages of 3.0 and 5.0 kV, and at magnifications of ×600 and ×700. The samples were detected with SE detectors.

### 3.11. Statistical Analysis

The results were obtained from at least three independent experiments carried out in duplicates and analyzed by single-factor ANOVA analyses. Quantitative results are presented as a mean *±* standard error. The difference between results was considered significant when the *p* value was less than 0.05. 

## 4. Conclusions

RB@ORMOSIL matrices immobilized onto linear low-density polyethylene can be prepared by an easy, cost-effective and simple thermal adhesion method. The prepared matrices exhibited high antibacterial activity and showed the low release of RB from the matrices in an aqueous environment. In the antibacterial tests, matrices prepared with a low APTES content showed higher activity than those with high APTES content, probably since the RB molecules on the matrix surface were more exposed to bacterial cells. The specimen RB6@OR@LLDPE can serve as an antibacterial surface for medical applications.

## Figures and Tables

**Table 1:** Characteristics of BL1, RB4 and RB6 matrices by BET analysis.

Sample	BET Surface Area, m^2^/g	Average Pore Volume, cm^3^/g	Average Pore Diameter, nm
BL1	311	2.8	3.6
RB4	279	3.0	3.7
RB6	225	2.2	4.1

**Table 2:** Leaching of RB from RB@ORMOSIL matrices.

Specimen *	Amount of Leached RB, µmol **	RB Leaching, %	Actual Loading of RB in Matrices, µmol/g
RB1	19.1	45.2	5.47
RB2	22.0	52.0	4.67
RB3	8.6	20.4	7.83
RB4	18.7	44.3	5.45
RB5	15.5	36.7	6.37
RB6	16.9	40.0	5.94

* In all the cases, the RB loading used for ORMOSIL synthesis was 0.073 mole % when calculated based on the total moles of silane precursors. ** Calculated on the basis of 42.2 µmol RB loaded onto the matrix.

**Table 3:** Conditions of RB@ORMOSIL matrix preparation.

Specimen	Molar Ratio TEOS:MTMOS:APTES	APTES, Mole % *	RB, 0.073 Mole % *
BL1	20.3:2.2:1	4.44	-
BL2	13.7:1.5:1	6.65	-
BL3	10.2:1.1:1	8.89	-
BL4	8.1:0.9:1	11.11	-
RB1	20.3:2.2:1	4.44	Immediately after the addition of APTES
RB2	13.7:1.5:1	6.65	Immediately after the addition of APTES
RB3	10.2:1.1:1	8.89	Immediately after the addition of APTES
RB4	8.1:0.9:1	11.11	Immediately after the addition of APTES
RB5	20.3:2.2:1	4.44	Before the addition of APTES
RB6	20.3:2.2:1	4.44	Parallel to the addition of APTES

* The content was calculated based on total moles of silane precursors.

## Data Availability

The data are contained within this publication.

## Abbreviations

APTES	(3-aminopropyl)triethoxsylane
BET	Brunauer–Emmett–Teller method
BG	Bioactive glass
BH	Brain–heart
BJH	Barrett−Joyner−Halenda
CFU	Colony-forming units
EtOH	Ethanol
HAIs	Healthcare-associated infections
LB	Luria Bertani
LLDPE	Linear low-density polyethylene
MIC	Minimal inhibitory concentration
MTMOS	Methyltrimethoxysilane
OD	Optical density
ORMOSIL	Organically modified silica
PACT	Photodynamic antimicrobial chemotherapy
PS	Photosensitizer
RB	Rose Bengal
ROS	Reactive oxygen species
SEM	Scanning electron microscope
TEOS	Tetraethylorthosilane
WHO	World Health Organization

## References

[1] Murphy F., Tchetchik A., Furxhi I. (2020). Reduction of Health Care-Associated Infections (HAIs) with Antimicrobial Inorganic Nanoparticles Incorporated in Medical Textiles: An Economic Assessment. Nanomaterials.

[2] Wylie M.P., Irwin N.J., Howard D., Heydon K., McCoy C.P. (2020). Hot-melt extrusion of photodynamic antimicrobial polymers for prevention of microbial contamination. J. Photochem. Photobiol. B Biol..

[3] Gouda M., Hebeish A. (2009). Preparation and Evaluation of CuO/Chitosan Nanocomposite for Antibacterial Finishing Cotton Fabric. J. Ind. Text..

[4] Ferreira A.M., Carmagnola I., Chiono V., Gentile P., Fracchia L., Ceresa C., Georgiev G., Ciardelli G. (2013). Surface modification of poly(dimethylsiloxane) by two-step plasma treatment for further grafting with chitosan–Rose Bengal photosensitizer. Surf. Coat. Technol..

[5] Griesser S., Jasieniak M., Vasilev K., Griesser H. (2021). Antimicrobial Peptides Grafted onto a Plasma Polymer Interlayer Platform: Performance upon Extended Bacterial Challenge. Coatings.

[6] Shi Q., Qian Z., Liu D., Liu H. (2017). Surface Modification of Dental Titanium Implant by Layer-by-Layer Electrostatic Self-Assembly. Front. Physiol..

[7] Escobar A., Muzzio N., Moya S.E. (2020). Antibacterial Layer-by-Layer Coatings for Medical Implants. Pharmaceutics.

[8] Muszanska A.K., Rochford E.T.J., Gruszka A., Bastian A.A., Busscher H.J., Norde W., Van Der Mei H.C., Herrmann A. (2014). Antiadhesive Polymer Brush Coating Functionalized with Antimicrobial and RGD Peptides to Reduce Biofilm Formation and Enhance Tissue Integration. Biomacromolecules.

[9] Cuervo-Rodríguez R., López-Fabal F., Gómez-Garcés J.L., Muñoz-Bonilla A., Fernández-García M. (2017). Contact Active Antimicrobial Coatings Prepared by Polymer Blending. Macromol. Biosci..

[10] Song J., Jang J. (2014). Antimicrobial polymer nanostructures: Synthetic route, mechanism of action and perspective. Adv. Colloid Interface Sci..

[11] Ahmed E.M. (2015). Hydrogel: Preparation, characterization, and applications: A review. J. Adv. Res..

[12] Tripathi V.S., Kandimalla V.B., Ju H. (2006). Preparation of ormosil and its applications in the immobilizing biomolecules. Sensors Actuators B Chem..

[13] Goh Y.-F., Alshemary A.Z., Akram M., Kadir M.R.A., Hussain R. (2014). Bioactive Glass: AnIn-VitroComparative Study of Doping with Nanoscale Copper and Silver Particles. Int. J. Appl. Glas. Sci..

[14] Trabelsi K., Ciriminna R., Albo Y., Pagliaro M. (2020). SilverSil: A New Class of Antibacterial Materials of Broad Scope. ChemistryOpen.

[15] Mahltig B., Soltmann U., Haase H. (2013). Modification of algae with zinc, copper and silver ions for usage as natural composite for antibacterial applications. Mater. Sci. Eng. C.

[16] Spagnul C., Turner L.C., Boyle R.W. (2015). Immobilized photosensitizers for antimicrobial applications. J. Photochem. Photobiol. B Biol..

[17] Nisnevitch M., Nakonechny F., Nitzan Y. (2010). Photodynamic antimicrobial chemotherapy by liposome-encapsulated water-soluble photosensitizers. Russ. J. Bioorg. Chem..

[18] Handoko Y.A., Rondonuwu F.S., Limantara L. (2015). The Photosensitizer Stabilities of Tookad^®^ on Aggregation, Acidification, and Day-light Irradiation. Procedia Chem..

[19] Konaté K., Mavoungou J.F., Lepengué A.N., Aworet-Samseny R.R., Hilou A., Souza A., Dicko M.H., M’Batchi B. (2012). Antibacterial activity against β- lactamase producing Methicillin and Ampicillin-resistants Staphylococcus aureus: Fractional Inhibitory Concentration Index (FICI) determination. Ann. Clin. Microbiol. Antimicrob..

[20] Cassidy C.M., Donnelly R., Elborn J., Magee N.D., Tunney M. (2012). Photodynamic Antimicrobial Chemotherapy (PACT) in combination with antibiotics for treatment of Burkholderia cepacia complex infection. J. Photochem. Photobiol. B Biol..

[21] Schäfer M., Schmitz C., Facius R., Horneck G., Milow B., Funken K.-H., Ortner J. (2007). Systematic Study of Parameters Influencing the Action of Rose Bengal with Visible Light on Bacterial Cells: Comparison Between the Biological Effect and Singlet-Oxygen Production. Photochem. Photobiol..

[22] Baptista M.S., Cadet J., Greer A., Thomas A.H. (2021). Photosensitization Reactions of Biomolecules: Definition, Targets and Mechanisms. Photochem. Photobiol..

[23] Cieplik F., Deng D., Crielaard W., Buchalla W., Hellwig E., Al-Ahmad A., Maisch T. (2018). Antimicrobial photodynamic therapy—What we know and what we don’t. Crit. Rev. Microbiol..

[24] Nakonechny F., Barel M., David A., Koretz S., Litvak B., Ragozin E., Etinger A., Livne O., Pinhasi Y., Gellerman G. (2019). Dark Antibacterial Activity of Rose Bengal. Int. J. Mol. Sci..

[25] Valkov A., Zinigrad M., Nisnevitch M. (2021). Photodynamic Eradication of *Trichophyton rubrum* and *Candida albicans*. Pathogens.

[26] Panzarini E., Inguscio V., Dini L. (2011). Overview of Cell Death Mechanisms Induced by Rose Bengal Acetate-Photodynamic Therapy. Int. J. Photoenergy.

[27] Vanerio N., Stijnen M., De Mol B.A., Kock L.M. (2019). Biomedical Applications of Photo- and Sono-Activated Rose Bengal: A Review. Photobiomodulat. Photomed. Laser Surg..

[28] Martins Estevão B., Cucinotta F., Hioka N., Cossi M., Argeri M., Paul G., Marchese L., Gianotti E. (2015). Rose Bengal incorporated in mesostructured silica nanoparticles: Structural characterization, theoretical modeling and singlet oxygen delivery. Phys. Chem. Chem. Phys..

[29] Guo Y., Rogelj S., Zhang P. (2010). Rose Bengal-decorated silica nanoparticles as photosensitizers for inactivation of gram-positive bacteria. Nanotechnology.

[30] Thommes M., Kaneko K., Neimark A.V., Olivier J.P., Rodriguez-Reinoso F., Rouquerol J., Sing K.S.W. (2015). Physisorption of gases, with special reference to the evaluation of surface area and pore size distribution (IUPAC Technical Report). Pure Appl. Chem..

[31] Alothman Z.A. (2012). A Review: Fundamental Aspects of Silicate Mesoporous Materials. Materials.

[32] Grosman A., Ortega C. (2008). Capillary Condensation in Porous Materials. Hysteresis and Interaction Mechanism without Pore Blocking/Percolation Process. Langmuir.

[33] Santos A.M.M., Vasconcelos W.L. (1999). Obtention of nanostructured silica glass by sol-gel process with incorporation of lead compounds. Mater. Res..

[34] Lenza R., Vasconcelos W. (2001). Preparation of silica by sol-gel method using formamide. Mater. Res..

[35] Zhang X.-X., Lin W., Zheng J., Sun Y., Xia B., Yan L., Jiang B. (2018). Insight into the Organic–Inorganic Hybrid and Microstructure Tailor Mechanism of Sol–Gel ORMOSIL Antireflective Coatings. J. Phys. Chem. C.

[36] Adhikary J., Meistelman M., Burg A., Shamir D., Meyerstein D., Albo Y. (2017). Reductive Dehalogenation of Monobromo- and Tribromoacetic Acid by Sodium Borohydride Catalyzed by Gold Nanoparticles Entrapped in Sol–Gel Matrices Follows Different Pathways. Eur. J. Inorg. Chem..

[37] Alarcon E.I., Poblete H., Roh H., Couture J.-F., Comer J., Kochevar I.E. (2017). Rose Bengal Binding to Collagen and Tissue Photobonding. ACS Omega.

[38] Nakonechny F., Pinkus A., Hai S., Yehosha O., Nitzan Y., Nisnevitch M. (2012). Eradication of Gram-Positive and Gram-Negative Bacteria by Photosensitizers Immobilized in Polystyrene. Photochem. Photobiol..

[39] Valkov A., Nakonechny F., Nisnevitch M. (2014). Polymer-Immobilized Photosensitizers for Continuous Eradication of Bacteria. Int. J. Mol. Sci..

[40] Valkov A., Raik K.A., Mualem-Sinai Y., Nakonechny F., Nisnevitch M. (2018). Water Disinfection by Immobilized Photosensitizers. Water.

[41] Semenova O., Kobzev D., Yazbak F., Nakonechny F., Kolosova O., Tatarets A., Gellerman G., Patsenker L. (2021). Unexpected effect of iodine atoms in heptamethine cyanine dyes on the photodynamic eradication of Gram-positive and Gram-negative pathogens. Dyes Pigm..

[42] Valkov A., Nakonechny F., Nisnevitch M. (2015). Antibacterial Properties of Rose Bengal Immobilized in Polymer Supports. Appl. Mech. Mater..

[43] del Valle C.A., Pérez-Laguna V., Resta I.M., Gavara R., Felip-León C., Miravet J.F., Rezusta A., Galindo F. (2020). A cost-effective combination of Rose Bengal and off-the-shelf cationic polystyrene for the photodynamic inactivation of Pseudomonas aeruginosa. Mater. Sci. Eng. C.

[44] Gavara R., de Llanos R., Pérez-Laguna V., del Valle C.A., Miravet J.F., Rezusta A., Galindo F. (2021). Broad-Spectrum Photo-Antimicrobial Polymers Based on Cationic Polystyrene and Rose Bengal. Front. Med..

[45] Nakonieczna J., Wolnikowska K., Ogonowska P., Neubauer D., Bernat A., Kamysz W. (2018). Rose Bengal-Mediated Photoinactivation of Multidrug Resistant Pseudomonas aeruginosa Is Enhanced in the Presence of Antimicrobial Peptides. Front. Microbiol..

[46] Kurosu M., Mitachi K., Yang J., Pershing E.V., Horowitz B.D., Wachter E.A., Lacey J.W., Ji Y., Rodrigues D.J. (2022). Antibacterial Activity of Pharmaceutical-Grade Rose Bengal: An Application of a Synthetic Dye in Antibacterial Therapies. Molecules.

[47] Gurianov Y., Nakonechny F., Albo Y., Nisnevitch M. (2020). LLDPE Composites with Nanosized Copper and Copper Oxides for Water Disinfection. Polymers.

[48] Meistelman M., Meyerstein D., Burg A., Shamir D., Albo Y. (2021). “Doing More with Less”: Ni(II)@ORMOSIL, a Novel Sol-Gel Pre-Catalyst for the Reduction of Nitrobenzene. Catalysts.

